# A nurse-led, telephone-based patient support program for improving adherence in patients with relapsing-remitting multiple sclerosis using interferon beta-1a: Lessons from a consumer-based survey on adveva^®^ PSP

**DOI:** 10.3389/fpsyg.2022.965229

**Published:** 2022-08-24

**Authors:** Serena Barello, Damiano Paolicelli, Roberto Bergamaschi, Salvatore Cottone, Alessandra D'Amico, Viviana Annibali, Andrea Paolillo, Caterina Bosio, Valentina Panetta, Guendalina Graffigna

**Affiliations:** ^1^EngageMinds HUB, Department of Psychology, Consumer, Food & Health Engagement Research Center, Università Cattolica del Sacro Cuore, Milan, Italy; ^2^Department of Basic Medical Sciences, Neurosciences and Sense Organs, University of Bari “Aldo Moro”, Bari, Italy; ^3^IRCCS Mondino Foundation, Pavia, Italy; ^4^UOC Neurologia con Stroke Unit ARNAS Civico, Palermo, Italy; ^5^Medical Affairs Department, Merck Serono S.p.A., Rome, Italy, An Affiliate of Merck KGaA, Darmstadt, Germany; ^6^EngageMinds HUB, Department of Psychology, Consumer, Food & Health Engagement Research Center, Università Cattolica del Sacro Cuore, Cremona, Italy; ^7^L'altrastatisticasrl, Consultancy & Training, Biostatistics Office, Rome, Italy

**Keywords:** patient support program, multiple sclerosis, treatment adherence, patient engagement, disease modifying therapies, patient reported outcomes, patient reported experience measures

## Abstract

**Background:**

Evidence suggests that organizational models that provide care interventions including patient support programs may increase patient adherence to multiple sclerosis (MS) therapies by providing tailored symptom management, informational support, psychological and/or social support, lifestyle changes, emotional adjustment, health education, and tailored coaching, thus improving patients' overall quality of life across the disease course.

**Objective:**

The main objective of this study was to describe MS patients' self-reported experience of a nurse-led, telephone-based PSP and to explore its potential role in improving disease and therapy management skills.

**Methods:**

Survey data were analyzed from a subset of patients relapsing–remitting MS (RRMS) using interferon beta-1a already registered in the adveva^®^ PSP from three Italian multiple sclerosis centers with a consolidated experience in RRMS disease, treatment management, and PSP programs.

**Results:**

In total, 244 patient data at baseline were analyzed, of which 115 had a follow-up of at least 6 months. Results from this study provide an early view into the role of this PSP in improving the patients reported overall experience regarding disease management and injectable therapy, thus potentially ameliorating treatment adherence and decreasing health care cost. Moreover, study findings confirm the role of providing a patient-focused support by addressing non-medication-related topics in the PSP consultations. Indeed, patients involved in the adveva^®^ PSP program reported a better psychological status in the follow up as demonstrated by an increased optimism regarding their future, tolerance of disease uncertainty, and their perceived ability to benefit from external help and social support (informal caregivers).

**Conclusions:**

As such, it is reasonable to conclude that the involvement in the adveva^®^ PSP and the PSP's assistance in guiding patients on proper treatment self-management techniques is of great value to patients as it might contribute to improving engagement in their health care journey in terms of perceived self-care skills, emotional coping toward the future and the unpredictability of the disease course and their general attitudes toward the injection itself, involving pain tolerance.

## Introduction

Multiple sclerosis (MS) is a chronic immune-mediated, inflammatory neurological disease of the central nervous system (CNS) (Pugliatti et al., 2006; AISM, 2021). The disease is responsible for demyelination and axonal loss in an unpredictable pattern and may result in a relapsing or progressive clinical path. According to the WHO/MSIF MS Atlas, the estimated number of people with MS in the world is 2.8 million in 2020 (AISM, 2021). MS causes a wide variety of symptoms including fatigue, weakness, sensory impairments, cognitive impairment, and depression (Lakin et al., 2021). The incidence is very different in the various countries and overall, 2.1 new cases/year per 100,000 inhabitants in the world, with more than 1,2 million of cases in Europe (AISM, 2021). Since 1980, many epidemiological studies have classified Italy as a high-risk country for MS, with the highest rates in the Sardinia island (AISM, 2021). According to the Italian MS patient organization (AISM) estimates, there are 126,000 patients in Italy with an incidence of 3,600 cases per year (AISM, 2021).

The most common clinical form is relapsing-remitting MS (RRMS), where patients usually present with a fluctuating disease course that is unpredictable and transiently remitting (Knowles et al., 2021). Disease-modifying therapies (DMDs) can partly control MS evolution and reduce the frequency of relapses. However, to achieve good clinical outcomes, patients need to be effectively adherent to therapies and to become fully engaged as active players in their own disease management (Barello and Graffigna, 2015, 2016; Rieckmann et al., 2015; Ganguli et al., 2016; Jenerette and Mayer, 2016; Lenz and Harms, 2020; Nicholas et al., 2020).

Poor adherence to DMDs has been correlated with an increased risk of relapse (Moccia et al., 2015) and emergency admissions (Paolicelli et al., 2016) in this patient population.

Good treatment adherence is dependent on a wide range of factors and interventions not only clinical but also psychosocial. In particular, the patient's ability to effectively adhere to treatment is linked to adequate patients behaviors (i.e., self-management and self-care skills) (McNulty et al., 2004) and a good knowledge of the disease and treatments (i.e., health literacy level) (McNulty et al., 2004), a sense of control over the disease course and therapies (self-efficacy in self-care) (Buja et al., 2021), good communication skills with the healthcare providers (self-efficacy in the communication with health providers) (McNulty et al., 2004), treatment experience (i.e., satisfaction with therapy), adjustment to the disease (i.e., tolerance of disease uncertainty, sense of hope) (McNulty et al., 2004), presence of supportive relationships (i.e., perceived social support) (Siegel et al., 2008).

Studies showed that supporting the patient in maintaining positive hope for the future allows him to feel more “empowered,” with positive effects on quality of life and adherence (Buja et al., 2021). Adherence is also significantly influenced by disease-related factors such as disability, illness duration, depression, and quality of life (Giovannetti et al., 2020). Psychological coping has proved to be crucially important for adjusting to the adaptive demands of chronic diseases, and in the last few years, it has received growing interest in MS (Keramat Kar et al., 2019). Indeed, literature shows that considering the psychosocial characteristics of the patients when assessing his/her status during therapy may allow customized support and improve treatment adherence (Heesen et al., 2014).

As suggested by a recent study (Moccia et al., 2019), MS treatment and management benefit from integrated patient-care (diagnosis, treatment and follow-up), with a balanced and coordinated integration of local and centralized services. In this scenario, individualized, patient-centered treatment support (i.e., patient support programs—PSP) may provide better education and lead to greater treatment adherence, helping to achieve optimal clinical and economic outcomes (Ganguli et al., 2016). In addition, PSPs have been proven to provide successful opportunities to patients to increase their perception of empowerment and well-being (Kohlmann et al., 2013). Evidence suggests that initiatives such as nurse-led telephone-based supportive interventions increase patient adherence to chronic therapies (Ganguli et al., 2016). In particular, they can provide symptom management, informational support, psychological and/or social scaffolding, lifestyle behavioral change suggestions, emotional adjustment, health education, and tailored coaching, and they can improve patients' overall quality of life across disease conditions (Chow and Wong, 2010; Stolic et al., 2010; Soon-Rim Suh and Lee, 2017). Telephone-based support can provide access to people in remote areas and has, therefore, become a standard method of providing education and advice to patients with chronic diseases (Greenberg, 2000; Overend et al., 2008). It is generally believed that telephone-based interventions hold promise for extending the supportive care provided to patients with MS (Tietjen and Breitenstein, 2017; Yeroushalmi et al., 2020).

Within this scenario, the main objective of this paper was to describe MS patients' self-reported experience of a nurse-led, telephone-based PSP (i.e., adveva^®^ PSP) and to explore its potential role in improving their disease and therapy management skills.

## Materials and methods

### Study design and PSP description

This explorative project featured a retrospective secondary analysis of patient reported experience data and was conducted based on the anonymized interactive data registered by the adveva^®^ PSP platform. Merck provides a free PSP, called adveva^®^, designed to help patients receiving REBIF^®^ injectable treatment to improve the quality of their treatment and outcomes. The Merck PSP adveva^®^ is integrated with a qualified team of professionals and multi-channel system (website, app, and toll-free number), to provide operational services, technical assistance, practical advice, information, and materials to support patients in adhering to therapy. Patients' adhesion to the program is spontaneous and regulated by General Data Protection Regulation (D.Lgs.n. 196/2003 and EU 2016/679).

### Methods and sample data

Data were collected by IQVIA Patient Solution S.p.A (i.e., the data processor, under the art. 28 of the GDPR) on behalf of Merck Serono S.p.A. (i.e., the data controller), as part of the adveva^®^ patient support program. Data collection occurred between May 2019 and December 2020 and patients spontaneously agreed to the use of their anonymized data for customer research purposes.

Anonymized data were then analyzed by the statistical service of Merck Serono from a subset of patients relapsing–remitting MS (RRMS) using interferon beta-1a already registered in the adveva^®^ PSP from 3 Italian multiple sclerosis centers with a consolidated experience on RRMS disease, treatment management and PSP programs.

Data were related to the spontaneous answers that patients gave in the proactive phone calls with the PSP call center at baseline and at >6 months follow-up. The phone call was conducted by a group of 3 *ad-hoc* trained nurses (each participant was called every time by the same nurse) and was based on a structured survey (Table 1) aimed at monitoring the patient experience with the PSP to identify possible patients' unmet needs. Specific questions were asked about:

- **self-management and self-care attitudes** (i.e., “*I always know what to do to manage my illness”; “I am confident that I can recognize when I need specialist medical care and when I can manage a health problem on my own”*);
- **health literacy level** (i.e., “*The goal of all the treatments I undergo is clear to me”; “I know the characteristics of my disease”; “I know where to find reliable information about my disease and treatments”*);
- **perceived self-efficacy in communication with the care team** (i.e., “*I find it easy to communicate with my doctor on issues related to my illness and my treatments”; “It is easy for me to ask my doctor about my illness and my treatments”*);
- **tolerance of disease uncertainty and adjustment to the disease** (i.e., “*I never know how I will feel, there are days when it is better and days when it is worse”; I can plan my future, even though I am not sure how my disease will evolve; “I consider illness a normal part of my daily life”; “I can reconcile the management of my illness with all the other activities of my daily life”*);
- **sense of hope** (i.e., “*I can have hope for my future despite my illness I am positive about my future”*);
- **perceived social support** (i.e., “*I can count on one person in particular to help me in the daily management of my illness”; “My family and friends support me emotionally”; “I turn to a patient association to find support in the management of my disease”*);
- **treatment satisfaction with therapy** (i.e., “*I am satisfied with the therapy that I have been prescribed”*).

**Table 1:** Survey items.

**Item number**	**Italian version**	**English translation** **(for publishing purposes)**
1	L'obiettivo della terapia a cui mi sottopongo mi è chiaro	The goal of the therapy I am undergoing is clear to me
2	Conosco le caratteristiche della mia condizione di salute	I know the characteristics of my health condition
3	So dove reperire informazioni affidabili sulla mia condizione di salute e sulla mia terapia	I know where to find reliable information on my health condition and my therapy
4	So sempre cosa fare per gestire la mia terapia	I always know what to do to manage my therapy
5	Sono autonomo nella gestione della mia terapia	I am autonomous in the management of my therapy
6	Sono certo di poter riconoscere quando ho bisogno di rivolgermi a uno specialista e quando invece posso gestire un problema di salute per conto mio	I am sure I can recognize when I need to see a specialist and when I can manage a health problem on my own
7	So quali domande fare al personale sanitario su tematiche relative alla mia terapia	I know what questions to ask healthcare professionals about issues related to my therapy
8	È facile per me fare domande al personale sanitario sulla mia condizione di salute e sulla mia terapia	It is easy for me to ask healthcare professionals questions about my health condition and therapy
9	Riesco a prevedere i miei sintomi, so quando mi sentirò meglio o peggio	I can predict my symptoms; I know when I will feel better or worse
10	Sono tendenzialmente ottimista sul mio futuro e sul mio stato di salute	I tend to be optimistic about my future and my state of health
11	Considero la gestione della terapia una parte normale della mia routine quotidiana	I consider managing therapy a normal part of my daily routine
12	Riesco ad avere speranza nel mio futuro, nonostante la mia malattia	I can have hope for my future, despite my illness
13	Riesco a pianificare il mio futuro pur non avendo chiaro come si evolverà la mia condizione di salute	I can plan my future even though I am not sure how my health condition will evolve
14	Posso contare su una persona in particolare che mi aiuta nella gestione pratica delle attività quotidiane	I can count on one specific person that help me in the practical management of daily activities
15	La mia famiglia e i miei amici mi supportano emotivamente	My family and friends support me emotionally
16	Mi rivolgo ad una associazione di pazienti per trovare supporto nella gestione della mia salute	I turn to a patient association to find support in managing my health
17	Sono soddisfatto della terapia che mi è stata prescritta	I am satisfied with the therapy that has been prescribed for me
18	Sono soddisfatto del dispositivo che utilizzo per la somministrazione della terapia (*se paziente de novo:* Ho fiducia nell'utilizzo di un dispositivo medico per assumere la mia terapia)	I am satisfied with the device I use to administer the therapy (*If new patient*: I am confident in using a medical device to take my therapy)
19	L'iniezione è dolorosa	The injection is painful
20	Dover fare l'iniezione è la parte peggiore della gestione della mia malattia	Having to inject is the worst part of managing my disease
21	L'iniezione mi fa paura	The injection scares me
22	Penso che l'iniezione sia la via di somministrazione ottimale per questa terapia	I think injection is the optimal route of administration for this therapy

The patients could answer each statement with a value from 1 to 5 (1 = Strongly disagree; 2 = Disagree; 3 = Neither agree nor disagree; 4 = Agree and 5 = Strongly agree). For analysis purposes, questions have been grouped according to the level of patients' agreement at baseline (i.e., “Agree,” the statements with a very low percentage of disagreement (<5%); Medium Agree, the statements with a 5–10% of disagreement; low agree, the statements with more than 10% of disagreement).

### Statistical analyses

Quantitative data (months from first therapy) are presented as the median and interquartile range (q1–q3), categorical data as absolute frequency and percentage.

Chi-squared test or Fisher's exact test, when needed, were used to compare categorical variables between the follow-up group and the non-follow-up group. Non-parametric tests by Mann Whitney were used to compare gender and the time from therapy initiation and the responses to individual statements between two groups.

Wilcoxon's test was used to assess the variation of responses to each statement between responses at baseline and at follow-up.

The Stata 16.1 software was used for all analyzes and a *p*-value < 0.05 was considered statistically significant.

## Results

### Baseline data

In total, 244 patient data at baseline were analyzed, of which 115 have a follow-up of at least 6 months.

Patients with follow-up were significantly younger than patients without follow-up. In patients with follow-up, there is also a higher percentage of males even if statistical significance is at the limit (see Table 2 for more details about the study sample).

**Table 2:** Sample characteristics at baseline in patients with and without follow-up.

		**Without FU**		**With FU**		
		***n***	***%***	***n***	***%***	***p***
*N*		129		115		
Data available (*n*, %)		106	82.2	115	100	
Males (*n*, %)		25	23.6	41	35.7	0.050
Age (*n*, %)					0.010
18–40		42	39.6	50	43.5	
41–55		42	39.6	57	49.6	
>55		22	20.8	8	7.0	
Months from first therapy					0.604
Median (Q1–Q3)		68.9	40.9–96.3	63	33.9–98.7	

The answer to each statement is not statistically different between the two groups except for item number 10 in which patients with follow-up are less optimisticthan the others. See Supplementary material 1 for more details.

### Psychosocial measures

**Figure 1:**
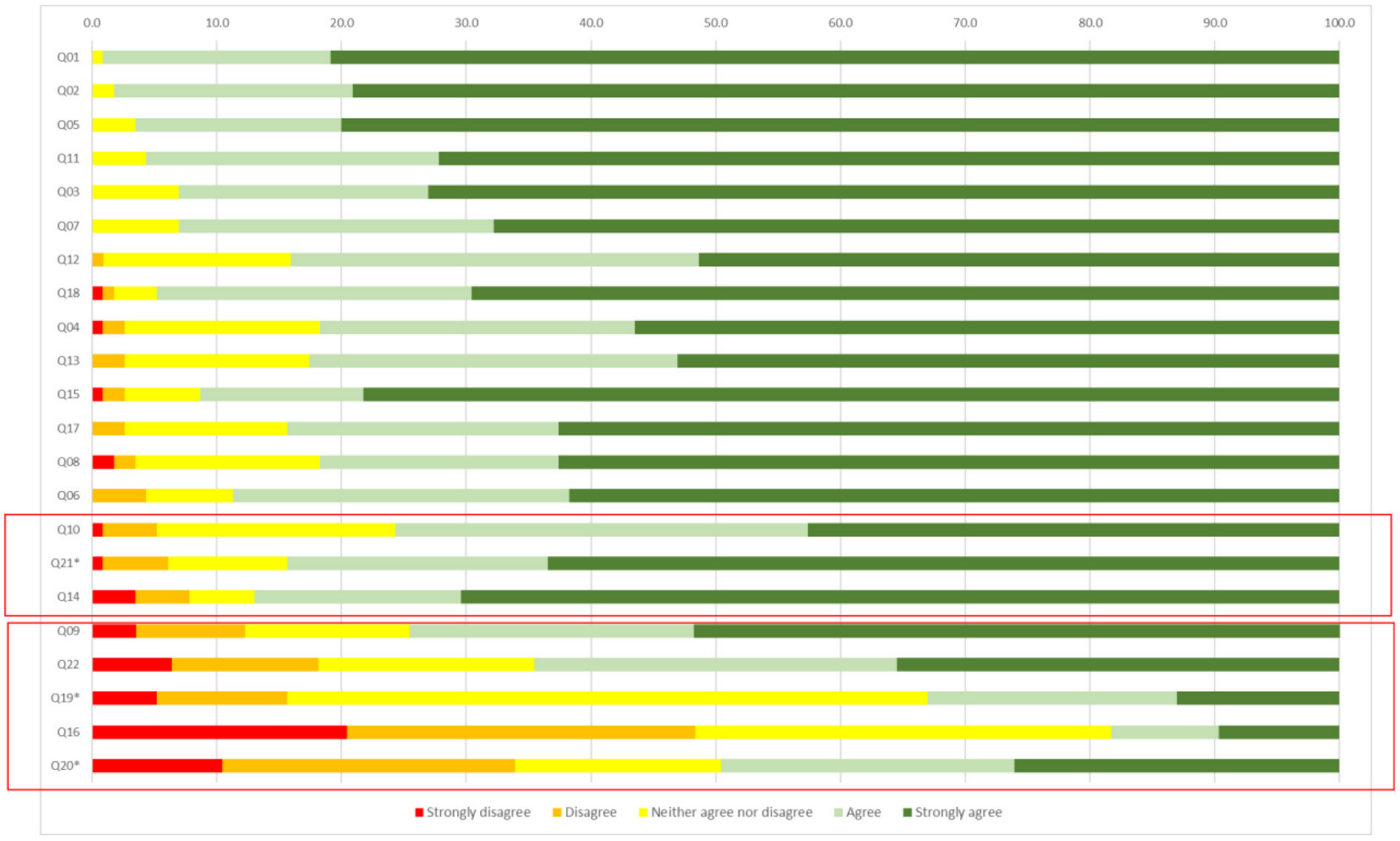
Patients' items' response distribution at baseline. *Are negative statements so answers were inverted.

Figure 1 shows the distribution of answers for each item of the 115 patients included in this study. Analysis was conducted by grouping items according to the level of patients' agreement/disagreement with them at baseline as follows:

- “*High agree items*”, the ones with a very low percentage of disagreement (<5%) (items: 1,8,11,13,15,17,18)
- “*Medium agree items*”, the ones with a 5%-10% of disagreement (items: 10, 14, 21)
- “*Low agree items*”, the ones with more than 10% of disagreement (Items: 9, 16, 19, 20, 22)

At baseline, 5.2% of patients disagreed with the item Q10 “*I tend to be optimistic about my future and my state of health*” and the 19% are neither agree nor disagree; moreover, the 7.8% of patients report having not a person who support them regularly (Q14) and the 6.1% of patients are afraid of the injection (Q21); the 12.3% of patients disagree with the item Q9: “*I can predict my symptoms, I know when I will feel better or worse*” and the 13.2% is neither agree or disagree.

The injection is painful (Q19) for 15.7% of patients and is the worst part of the disease (Q20) for the 33.9%. 18.2% of the participants disagree that the injection is the optimal way of administration (Q22).

Comparing responses between male and female participants, there is a higher percentage of men who are optimistic about the future and their state of health (Q10), and who do not consider the injection painful (Q19). See Supplementary material 2 for more details about differences at baseline between male and female responders.

### Measures at follow-up

For every item (out of item number 19, that is: “*The injection is painful”*) there is a significant change between the two-time points (see Appendix 1 for details about each item). The percentage of patients' disagreement in QD10 (“*I tend to be optimistic about my future and my state of health”)* decreases from 5.2 to 1.8%.

Out of 7 patients who are afraid of injection, only 2 are still agree with Q21, but the percentage of agreeing and strongly agree is the same in the two-time points because there are 4 patients who disagreed (or neither agree or disagree) with the statement at baseline but are agree at follow up.

At follow-up, there are patients who disagree with item 9 (“*I can predict my symptoms; I know when I will feel better or worse*”) and only 4.4% agree or disagree. The percentage of patients who considered the injection painful and the worst part of the disease decreased from 15.7 to 9.6%, and from 33.9 to 19.1%, respectively.

The percentage of patients who disagree that the injection is the optimal way of administration decreased from 18.2 to 7%. The level of patient agreement with more than 90% of items increased significantly comparing responses at baseline and follow-up. Overall, 58% of patients increased their level of agreement at least for 19 items out of 22.

**Figure 2:**
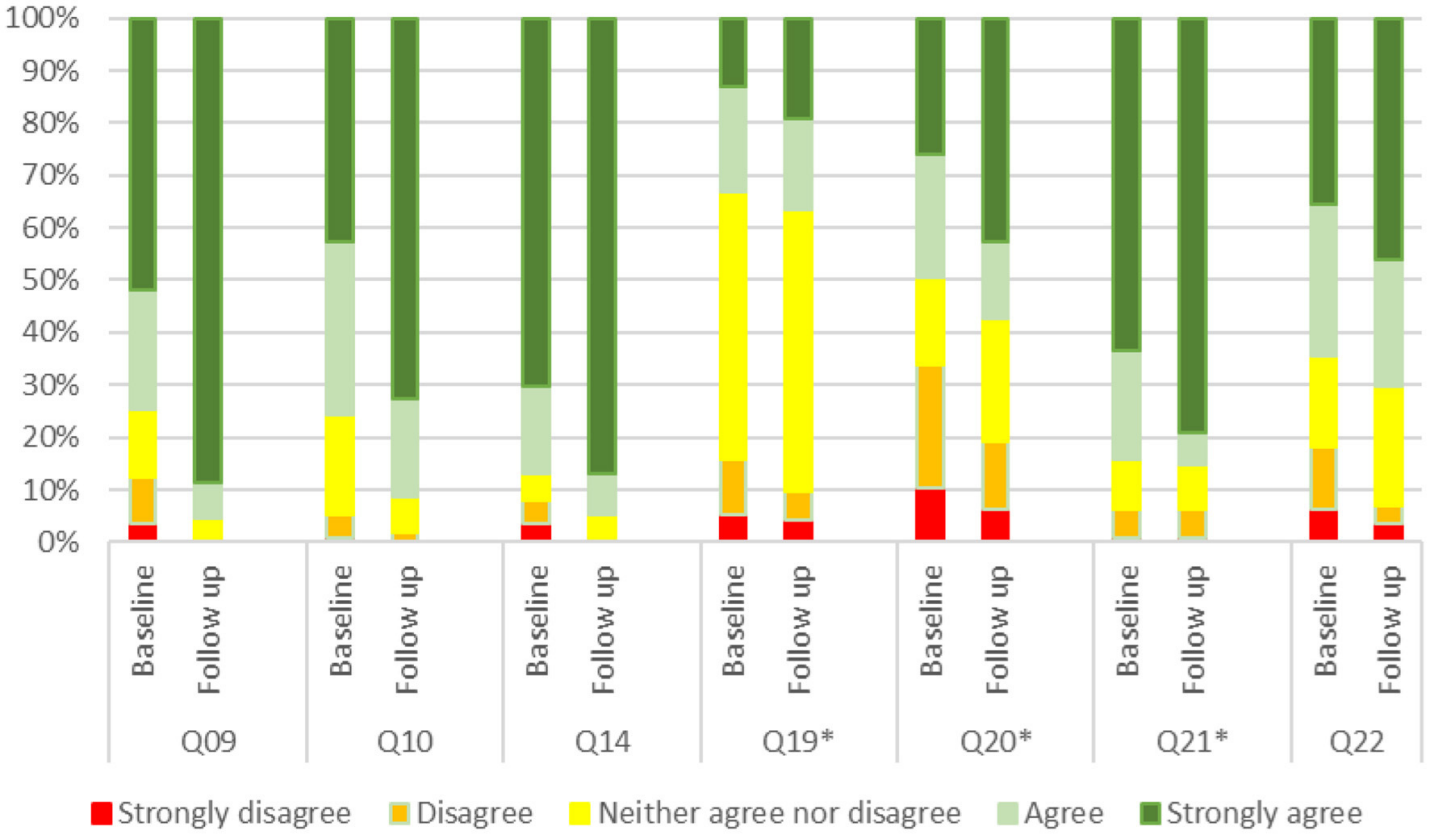
Most significant change in items' response between baseline and follow-up. *Are negative statements so answers were inverted.

Figure 2 describes items that reported the most significant change between baseline and follow-up.

## Discussion

The adveva^®^ PSP assessed in this pilot study is a patient-centered, holistic program that was designed to improve the overall care experience of patients diagnosed with relapsing-remitting multiple sclerosis and its therapeutic management. It provides a broad range of resources to support patients throughout their disease treatment, including standardized phone-based contact with *ad hoc* trained nurses. The current results provide an early view into the role of this PSP in improving the patients reported overall experience regarding disease management and therapy, thus potentially ameliorating treatment adherence and decreasing health care cost. This study adds to the body of evidence examined in a previous study that targeted a systematic review of the impact of PSPs on patient outcomes (Ganguli et al., 2016). While comprehensive, this review reported only a few studies of the effect of PSPs involving patients diagnosed with multiple sclerosis. The current results are in line with those of other general disease therapy management programs for chronic patients receiving injectable treatment, which found improvements in the self-reported experience of disease management (Stockl et al., 2010; Kohlmann et al., 2013; Van den Bosch et al., 2017).

In particular, one of the patient experience domains that appear to be more impacted by the adveva^^®^^ PSP attendance is the one related to the ability of the patients to self-manage their disease and treatment, as testified by the increased endorsement of statements like “*I can predict my symptoms; I know when I will feel better or worse*” when comparing baseline with follow-up measures. This result is generally consistent with those of previous studies examining the impact of similar initiatives. For example, participation in nurse-led, consultation-based interventions has been associated with significant improvements in disease-specific knowledge, self-efficacy skills, and health perceptions (Mohr et al., 2005).

On the other hand, the patient-reported, experience with injection significantly improves when comparing baseline with follow-up data: injection is perceived painful and scary and, at the same time, patients reported with a higher frequency that injection is the optimal route of administration for their therapy. Literature suggests that patients' attitudes about their treatments and disease, in particular the extent to which they can exert some sort of control over their disease course, can contribute to moderating the pain experience itself (DePalma and Weisse, 1997).

Problems with and fears of injections and perceived lack of efficacy are considered major barriers to sustained adherence among patients with MS (Moccia et al., 2015). The more positive experience about these issues collected in the follow-up may indicate a positive influence of the program on patients' behavior, as suggested by previous studies indicating the relevance of emphasizing the patient's central role in managing their health and treatment (Costello et al., 2008; Jones et al., 2013).

Moreover, this study's findings confirm the role of providing a patient-focused support by addressing non-medication-related topics in the PSP consultations. Indeed, the patients involved in the adveva^^®^^ PSP program reported a better psychological status in the follow up as demonstrated by an increased optimism regarding their own future, tolerance of disease uncertainty, and their perceived ability to benefit from external help and social support (informal caregivers). Therefore, this program contributes to making a further step toward a paradigm shift to organizational models focused on patient engagement and person-centered care, as recommended by international health policies (Moss et al., 2010; World Health Organization, 2015; Triberti and Barello, 2016; Graffigna et al., 2020). Moreover, considering the new treatment scenario and barriers that MS patients may experience in accessing specialty care for evaluation and treatment, our results might support the relevance of telephone-based PSP—such as the one described in this work—as a potential bridge to close the access gap for this clinical population. Moreover, this model of care may guarantee more equitable care for hard-to-reach or more vulnerable populations: many countries only have treatment services in a few major cities, and access to professional clinical and supportive services for people in regional and remote areas is a challenge. Moreover, it is possible to hypothesize that personal attention received from an experienced nurse seems to positively influence patient satisfaction, as confirmed by other studies (Moss et al., 2010; Barello and Graffigna, 2015; Triberti and Barello, 2016; Cui et al., 2019; Lotfi et al., 2019; Meier et al., 2019).

## Limitations

Although the results of the present study help us to better understand the role of PSP in improving MS patients' adherence, the limitations of this study, such as a short-term follow-up time, and limited and not representative sample size should also be considered. Larger, longer-term, and methodologically stronger studies (i.e., RCT) are needed to further validate our present findings in this clinical population. Moreover, as in any observational study, only observed confounders were accounted for, hence the findings of the study should be interpreted with the caution of unobserved confounding.

Moreover, due to the high level of agreement with most statements at baseline, it is possible to assume that patients involved in the PSP are affected by self-selection bias (i.e., the ones more engaged and adherent might tend to participate most in this kind of initiative). Finally, one major limitation is the relatively low number of cases during follow-up, probably due to the COVID-19 pandemic which occurred during data collection and may have impacted the validity of the results.

The authors of this work acknowledge these limitations and hope that this initial, pilot research will provide a proof of concept to foster future research to use more sophisticated sampling strategies, including a randomized or matched control group design to test the effectiveness of such intervention on patient's adherence and clinical outcomes to infer causality behind the associations reported here. Moreover, future studies should include an objective measure of clinical outcomes besides patient-reported ones. In addition, it will be important to validate these findings in other countries to understand if these findings are specific to Italian patients or can be generalized to a broader patient pool. Finally, research examining the contributions of specific PSP components to adherence, outcomes, and cost is warranted and planned.

## Conclusion

In this study, patients provide early insights about the impact of MS patient enrollment in this PSP on disease and therapy management experience. Enrollment in the PSP was associated with a more positive illness experience. As such, it is reasonable to conclude that the involvement in the adveva^^®^^ PSP and, in particular, the PSP's assistance in guiding patients on proper treatment self-management techniques is of great value to patients as it might contribute to improve engagement in their health care journey in terms of perceived self-care skills, emotional coping toward the future and the unpredictability of the disease course and their general attitudes toward the injection itself, involving pain tolerance. These data provide support for prescribing physicians to encourage enrollment in PSPs for MS conditions and pharmaceutical companies to further develop and invest in multifaceted PSPs.

## Data availability statement

The raw data supporting the conclusions of this article will be made available by the authors, without undue reservation.

## Ethics statement

Ethical review and approval was not required for the study on human participants in accordance with the local legislation and institutional requirements. The patients/participants provided their written informed consent to participate in this study.

## Author contributions

SB, CB, and GG drafted the manuscript, contributed study concept, and design. CB, DP, RB, and SC collected the data. VP analyzed the data. GG, DP, RB, AD'A, VA, AP, VP, and SC critically revised the manuscript. All authors contributed to data interpretation and have read and approved the final submitted version of the manuscript.

## Funding

Publication development assistance was provided by EngageMinds HUB, Consumer, Food and Health Engagement Research Center, Department of Psychology, Università Cattolica del Sacro Cuore, Milan and Cremona, Italy, and funded by Merck Serono S.p.A., Rome, Italy, an affiliate of Merck KGaA, Darmstadt, Germany (CrossRef Funder ID: 10.13039/100009945). The authors declare that this study received funding from Merck Serono S.p.A., Rome. The funder was not involved in the study design, collection, analysis, interpretation of data, the writing of this article.

## Conflict of interest

Authors VA, AD'A, and AP contributed to this work as employees of Merck Serono S.p.A., Rome. VP contributed to this work as an employee of L'altrastatisticasrl, Consultancy & Training, Biostatistics Office, Rome. The remaining authors declare that the research was conducted in the absence of any commercial or financial relationships that could be construed as a potential conflict of interest.

## Publisher's note

All claims expressed in this article are solely those of the authors and do not necessarily represent those of their affiliated organizations, or those of the publisher, the editors and the reviewers. Any product that may be evaluated in this article, or claim that may be made by its manufacturer, is not guaranteed or endorsed by the publisher.

## References

[B1] AISM (2021). Barometro Della Sclerosi Multipla. Available online at: https://agenda.aism.it/2021/index.php.

[B2] Barello S. Graffigna G. (2015). Patient engagement in healthcare: pathways for effective medical decision making. Neuropsychol. Trends 17, 53–65. 10.7358/neur-2015-017-bare

[B3] Barello S. Graffigna G. (2016). “Engagement-sensitive decision making: training doctors to sustain patient engagement in medical consultations,” in Patient Engagement: A Consumer-Centered Model to Innovate Healthcare, eds G. Graffigna, S. Barello, and S. Tribeti (Warsav: De Gruyter), 78–93.

[B4] Buja A. Graffigna G. Mafrici S. F. Baldovin T. Pinato C. Bolzonella U.. (2021). Adherence to therapy, physical and mental quality of life in patients with multiple sclerosis. J. Pers. Med. 11:672. 10.3390/jpm11070672 34357139 PMC8303119

[B5] Chow S. K. Y. Wong F. K. (2010). Health-related quality of life in patients undergoing peritoneal dialysis: effects of a nurse-led case management programme. J. Adv. Nurs. 66, 1780–1792. 10.1111/j.1365-2648.2010.05324.x 20557392

[B6] Costello K. Kennedy P. Scanzillo J. O. (2008). Recognizing nonadherence in patients with multiple sclerosis and maintaining treatment adherence in the long term. Medscape J. Med. 10:225. 19008986 PMC2580090

[B7] Cui X. Zhou X. Ma L. L. Sun T. W. Bishop L. Gardiner F. W.. (2019). A nurse-led structured education program improves self-management skills and reduces hospital readmissions in patients with chronic heart failure: a randomized and controlled trial in China. Rural Remote Health. 19, 47–54. 10.22605/RRH5270 31113205

[B8] DePalma M. T. Weisse C. S. (1997). Psychological influences on pain perception and non-pharmacologic approaches to the treatment of pain. J. Hand Ther. 10, 183–191. 10.1016/S0894-1130(97)80072-5 9188037

[B9] Ganguli A. Clewell J. Shillington A. C. (2016). The impact of patient support programs on adherence, clinical, humanistic, and economic patient outcomes: a targeted systematic review. Patient Prefer. Adher. 10:711. 10.2147/PPA.S101175 27175071 PMC4854257

[B10] Giovannetti A. M. Barabasch A. Giordano A. Quintas R. Barello S. Graffigna G.. (2020). Construction of a user-led resource for people transitioning to secondary progressive multiple sclerosis: results of an international nominal group study. Front. Neurol. 11:798. 10.3389/fneur.2020.00798 33013615 PMC7461961

[B11] Graffigna G. Barello S. Riva G. Corbo M. Damiani G. Iannone P.. (2020). Italian consensus statement on patient engagement in chronic care: process and outcomes. Int. J. Environ. Res. Public Health 17:4167. 10.3390/ijerph17114167 32545278 PMC7312656

[B12] Greenberg M. E.. (2000). Telephone nursing: evidence of client and organizational benefits. Nurs. Econ. 18, 117–123. 11052013

[B13] Heesen C. Bruce J. Feys P. Sastre-Garriga J. Solari A. Eliasson L.. (2014). Adherence in multiple sclerosis (ADAMS): classification, relevance, and research needs. A meeting report. Multiple Scler. J. 20, 1795–1798. 10.1177/1352458514531348 24756569

[B14] Jenerette C. M. Mayer D. K. (2016). Patient-provider communication: the rise of patient engagement. Semin. Oncol. Nurs. 32, 134–143. 10.1016/j.soncn.2016.02.007 27137470

[B15] Jones J. L. Scheidt D. J. Kaushal R. S. Carroll C. A. (2013). Assessing the role of patient support services on adherence rates in patients using glatiramer acetate for relapsing-remitting multiple sclerosis. J. Med. Econ. 16, 213–220. 10.3111/13696998.2012.744316 23098539

[B16] Keramat Kar M. Whitehead L. Smith C. M. (2019). Characteristics and correlates of coping with multiple sclerosis: a systematic review. Disabil. Rehabil. 41, 250–264. 10.1080/09638288.2017.1387295 28994622

[B17] Knowles L. M. Esselman E. C. Turner A. P. Phillips K. M. Herring T. E. Alschuler K. N.. (2021). Depressive symptoms and suicidal ideation in progressive multiple sclerosis compared with relapsing-remitting multiple sclerosis: results from a cross-sectional survey. Arch. Phys. Med. Rehabil. 102, 694–701. 10.1016/j.apmr.2020.09.385 33080210

[B18] Kohlmann T. Wang C. Lipinski J. Hadker N. Caffrey E. Epstein M.. (2013). The impact of a patient support program for multiple sclerosis on patient satisfaction and subjective health status. J. Neurosci. Nurs. 45, E3–E14. 10.1097/JNN.0b013e31828a4161 23636073

[B19] Lakin L. Davis B. E. Binns C. C. Currie K. M. Rensel M. R. (2021). Comprehensive approach to management of multiple sclerosis: addressing invisible symptoms—a narrative review. Neurol. Ther. 10, 75–98. 10.1007/s40120-021-00239-2 33877583 PMC8057008

[B20] Lenz F. Harms L. (2020). The impact of patient support programs on adherence to disease-modifying therapies of patients with relapsing-remitting multiple sclerosis in germany: a non-interventional, prospective study. Adv. Ther. 37, 2999–3009. 10.1007/s12325-020-01349-3 32333326 PMC7467433

[B21] Lotfi M. Zamanzadeh V. Valizadeh L. Khajehgoodari M. (2019). Assessment of nurse–patient communication and patient satisfaction from nursing care. Nurs. Open 6, 1189–1196. 10.1002/nop2.316 31367445 PMC6650658

[B22] McNulty K. Livneh H. Wilson L. M. (2004). Perceived uncertainty, spiritual well-being, and psychosocial adaptation in individuals with multiple sclerosis. Rehabil. Psychol. 49:91. 10.1037/0090-5550.49.2.91

[B23] Meier A. Erickson J. I. Snow N. Kline M. (2019). Nurse and patient satisfaction. JONA J. Nurs. Administr. 49, 520–522. 10.1097/NNA.0000000000000814 31651609

[B24] Moccia M. Palladino R. Russo C. Massarelli M. Nardone A. Triassi M.. (2015). How many injections did you miss last month? A simple question to predict interferon β-1a adherence in multiple sclerosis. Expert Opin. Drug Deliv. 12, 1829–1835. 10.1517/17425247.2015.1078789 26371561

[B25] Moccia M. Tajani A. Acampora R. Signoriello E. Corbisiero G. Vercellone A.. (2019). Healthcare resource utilization and costs for multiple sclerosis management in the Campania region of Italy: comparison between centre-based and local service healthcare delivery. PLoS ONE 14:e0222012. 10.1371/journal.pone.0222012 31536513 PMC6752775

[B26] Mohr D. C. Burke H. Beckner V. Merluzzi N. (2005). A preliminary report on a skills-based telephone-administered peer support programme for patients with multiple sclerosis. Multiple Scler. J. 11, 222–226. 10.1191/1352458505ms1150oa 15795964

[B27] Moss A. C. Chaudhary N. Tukey M. Junior J. Cury D. Falchuk K. R.. (2010). Impact of a patient-support program on mesalamine adherence in patients with ulcerative colitis—a prospective study. J. Crohn's Colitis 4, 171–175. 10.1016/j.crohns.2009.10.002 21122501

[B28] Nicholas J. A. Edwards N. C. Edwards R. A. Dellarole A. Grosso M. Phillips A. L. (2020). Real-world adherence to, and persistence with, once-and twice-daily oral disease-modifying drugs in patients with multiple sclerosis: a systematic review and meta-analysis. BMC Neurol. 20, 1–15. 10.1186/s12883-020-01830-0 32664928 PMC7371467

[B29] Overend A. Khoo K. Delorme M. Krause V. Avanessian A. Saltman D. (2008). Evaluation of a nurse-led telephone follow-up clinic for patients with indolent and chronic hematological malignancies: a pilot study. Can. Oncol. Nurs. J. 18, 64–68. 10.5737/1181912x1826468 18649698

[B30] Paolicelli D. Cocco E. Di Lecce V. Direnzo V. Moiola L. Lanzillo R.. (2016). Exploratory analysis of predictors of patient adherence to subcutaneous interferon beta-1a in multiple sclerosis: TRACER study. Expert Opin. Drug Deliv. 13, 799–805. 10.1517/17425247.2016.1158161 26922837

[B31] Pugliatti M. Rosati G. Carton H. Riise T. Drulovic J. Vécsei L.. (2006). The epidemiology of multiple sclerosis in Europe. Eur. J. Neurol. 13, 700–722. 10.1111/j.1468-1331.2006.01342.x 16834700

[B32] Rieckmann P. Boyko A. Centonze D. Elovaara I. Giovannoni G. Havrdová E.. (2015). Achieving patient engagement in multiple sclerosis: a perspective from the multiple sclerosis in the 21st Century Steering Group. Mult. Scler. Relat. Disord. 4, 202–218. 10.1016/j.msard.2015.02.005 26008937

[B33] Siegel S. D. Turner A. P. Haselkorn J. K. (2008). Adherence to disease-modifying therapies in multiple sclerosis: does caregiver social support matter?. Rehabil. Psychol. 53:73. 10.1037/0090-5550.53.1.73

[B34] Soon-Rim Suh R. N. Lee M. K. (2017). Effects of nurse-led telephone-based supportive interventions for patients with cancer: a meta-analysis. Oncol. Nurs. Forum 44:E168. 10.1188/17.ONF.E168-E184 28632251

[B35] Stockl K. M. Shin J. S. Gong S. Harada A. S. Solow B. K. Lew H. C. (2010). Improving patient self-management of multiple sclerosis through a disease therapy management program. Am. J. Manag. Care 16, 139–144. 20148619

[B36] Stolic S. Mitchell M. Wollin J. (2010). Nurse-led telephone interventions for people with cardiac disease: a review of the research literature. Eur. J. Cardiovasc. Nurs. 9, 203–217. 10.1016/j.ejcnurse.2010.02.003 20381427

[B37] Tietjen K. M. Breitenstein S. (2017). A nurse-led telehealth program to improve emotional health in individuals with multiple sclerosis. J. Psychosoc. Nurs. Ment. Health Serv. 55, 31–37. 10.3928/02793695-20170301-04 28287673

[B38] Triberti S. Barello S. (2016). The quest for engaging AmI: patient engagement and experience design tools to promote effective assisted living. J. Biomed. Inform. 63, 150–156. 10.1016/j.jbi.2016.08.010 27515924

[B39] Van den Bosch F. Ostor A. J. Wassenberg S. Chen N. Wang C. Garg V.. (2017). Impact of participation in the adalimumab (Humira) patient support program on rheumatoid arthritis treatment course: results from the PASSION study. Rheumatol. Ther. 4, 85–96. 10.1007/s40744-017-0061-7 28361468 PMC5443730

[B40] World Health Organization (2015). WHO Global Strategy on People-Centred Integrated Health Services: Interim Report Service Delivery and Safety.

[B41] Yeroushalmi S. Maloni H. Costello K. Wallin M. T. (2020). Telemedicine and multiple sclerosis: a comprehensive literature review. J. Telemed. Telecare 26, 400–413. 10.1177/1357633X19840097 31042118

