# Supplementary material for: A nurse-led, telephone-based patient support program for improving adherence in patients with relapsing-remitting multiple sclerosis using interferon beta-1a: Lessons from a consumer-based survey on adveva® PSP
Source: Front Psychol. 2022 Aug 24;13:965229. doi: 10.3389/fpsyg.2022.965229 (PMC9454016; doi:10.3389/fpsyg.2022.965229)
Supplement: Supplementary file 1 [file Table_1.docx]

**Supplementary materials**

**Table 1. Changes from baseline to follow-up for each survey item**

|  |  | Baseline | | | Follow up | | | | p value | |
| --- | --- | --- | --- | --- | --- | --- | --- | --- | --- | --- |
|  |  | **n** | **%** | | **n** | | **%** | |  | |
| Q1 |  |  |  | |  | |  | | <0.001 | |
|  | **NR** |  |  | |  | |  | |  | |
|  | **Strongly disagree** |  | 0.0 | |  | | 0.0 | |  | |
|  | **Disagree** |  | 0.0 | |  | | 0.0 | |  | |
|  | **Neither agree nor disagree** | 1 | 0.9 | |  | | 0.0 | |  | |
|  | **Agree** | 21 | 18.3 | | 2 | | 1.7 | |  | |
|  | **Strongly agree** | 93 | 80.9 | | 113 | | 98.3 | |  | |
|  |  |  |  | |  | |  | |  | |
|  | **Median (q1-q3)** | 5 | 5-5 | | 5 | | 5-5 | |  | |
| Q2 |  |  |  | |  | |  | | <0.001 | |
|  | **NR** |  |  | |  | |  | |  | |
|  | **Strongly disagree** | 0 | 0.0 | |  | | 0.0 | |  | |
|  | **Disagree** | 0 | 0.0 | |  | | 0.0 | |  | |
|  | **Neither agree nor disagree** | 2 | 1.7 | | 1 | | 0.9 | |  | |
|  | **Agree** | 22 | 19.1 | | 3 | | 2.6 | |  | |
|  | **Strongly agree** | 91 | 79.1 | | 111 | | 96.5 | |  | |
|  |  |  |  | |  | |  | |  | |
|  | **Median (q1-q3)** | 5 | 5-5 | | 5 | | 5-5 | |  | |
| Q3 |  |  |  | |  | |  | | <0.001 | |
|  | **NR** |  | |  | |  | |  | |  |
|  | **Strongly disagree** | 0 | | 0.0 | |  | | 0.0 | |  |
|  | **Disagree** | 0 | | 0.0 | |  | | 0.0 | |  |
|  | **Neither agree nor disagree** | 8 | | 7.0 | | 2 | | 1.7 | |  |
|  | **Agree** | 23 | | 20.0 | | 5 | | 4.3 | |  |
|  | **Strongly agree** | 84 | | 73.0 | | 108 | | 93.9 | |  |
|  |  |  | |  | |  | |  | |  |
|  | **Median (q1-q3)** | 5 | | 4-5 | | 5 | | 5-5 | |  |
| Q4 |  |  | |  | |  | |  | | <0.001 |
|  | **NR** |  | |  | |  | |  | |  |
|  | **Strongly disagree** | 1 | | 0.9 | |  | | 0.0 | |  |
|  | **Disagree** | 2 | | 1.7 | |  | | 0.0 | |  |
|  | **Neither agree nor disagree** | 18 | | 15.7 | | 2 | | 1.7 | |  |
|  | **Agree** | 29 | | 25.2 | | 9 | | 7.8 | |  |
|  | **Strongly agree** | 65 | | 56.5 | | 104 | | 90.4 | |  |
|  |  |  | |  | |  | |  | |  |
|  | **Median (q1-q3)** | 5 | | 4-5 | | 5 | | 5-5 | |  |
| Q5 |  |  | |  | |  | |  | | 0.001 |
|  | **NR** | 0 | |  | | 1 | |  | |  |
|  | **Strongly disagree** |  | |  | |  | |  | |  |
|  | **Disagree** |  | |  | |  | |  | |  |
|  | **Neither agree nor disagree** | 4 | | 3.5 | | 3 | | 2.6 | |  |
|  | **Agree** | 19 | | 16.5 | | 1 | | 0.9 | |  |
|  | **Strongly agree** | 92 | | 80.0 | | 110 | | 96.5 | |  |
|  |  |  | |  | |  | |  | |  |
|  | **Median (q1-q3)** | 5 | | 5-5 | | 5 | | 5-5 | |  |
| Q6 |  |  | |  | |  | |  | | <0.001 |
|  | **NR** |  | |  | |  | |  | |  |
|  | **Strongly disagree** | 0 | | 0.0 | |  | |  | |  |
|  | **Disagree** | 5 | | 4.3 | |  | |  | |  |
|  | **Neither agree nor disagree** | 8 | | 7.0 | | 2 | | 1.7 | |  |
|  | **Agree** | 31 | | 27.0 | | 8 | | 7.0 | |  |
|  | **Strongly agree** | 71 | | 61.7 | | 105 | | 91.3 | |  |
|  |  |  | |  | |  | |  | |  |
|  | **Median (q1-q3)** | 5 | | 4-5 | | 5 | | 5-5 | |  |
| Q7 |  |  | |  | |  | |  | | <0.001 |
|  | **NR** | 0 | |  | | 1 | |  | |  |
|  | **Strongly disagree** |  | |  | |  | |  | |  |
|  | **Disagree** |  | |  | |  | |  | |  |
|  | **Neither agree nor disagree** | 8 | | 7.0 | | 1 | | 0.9 | |  |
|  | **Agree** | 29 | | 25.2 | | 3 | | 2.6 | |  |
|  | **Strongly agree** | 78 | | 67.8 | | 110 | | 96.5 | |  |
|  |  |  | |  | |  | |  | |  |
|  | **Median (q1-q3)** | 5 | | 4-5 | | 5 | | 5-5 | |  |
| Q8 |  |  | |  | |  | |  | | <0.001 |
|  | **NR** | 0 | |  | | 1 | |  | |  |
|  | **Strongly disagree** | 2 | | 1.7 | |  | |  | |  |
|  | **Disagree** | 2 | | 1.7 | |  | |  | |  |
|  | **Neither agree nor disagree** | 17 | | 14.8 | | 4 | | 3.5 | |  |
|  | **Agree** | 22 | | 19.1 | | 4 | | 3.5 | |  |
|  | **Strongly agree** | 72 | | 62.6 | | 106 | | 93.0 | |  |
|  |  |  | |  | |  | |  | |  |
|  | **Median (q1-q3)** | 5 | | 4-5 | | 5 | | 5-5 | |  |
| Q9 |  |  | |  | |  | |  | | <0.001 |
|  | **NR** | 1 | |  | | 2 | |  | |  |
|  | **Strongly disagree** | 4 | | 3.5 | |  | |  | |  |
|  | **Disagree** | 10 | | 8.8 | |  | |  | |  |
|  | **Neither agree nor disagree** | 15 | | 13.2 | | 5 | | 4.4 | |  |
|  | **Agree** | 26 | | 22.8 | | 8 | | 7.1 | |  |
|  | **Strongly agree** | 59 | | 51.8 | | 100 | | 88.5 | |  |
|  |  |  | |  | |  | |  | |  |
|  | **Median (q1-q3)** | 5 | | 3-5 | | 5 | | 5-5 | |  |
| Q10 |  |  | |  | |  | |  | | <0.001 |
|  | **NR** | 0 | |  | | 1 | |  | |  |
|  | **Strongly disagree** | 1 | | 0.9 | |  | |  | |  |
|  | **Disagree** | 5 | | 4.3 | | 2 | | 1.8 | |  |
|  | **Neither agree nor disagree** | 22 | | 19.1 | | 8 | | 7.0 | |  |
|  | **Agree** | 38 | | 33.0 | | 21 | | 18.4 | |  |
|  | **Strongly agree** | 49 | | 42.6 | | 83 | | 72.8 | |  |
|  |  |  | |  | |  | |  | |  |
|  | **Median (q1-q3)** | 4 | | 4-5 | | 5 | | 4-5 | |  |
| Q11 |  |  | |  | |  | |  | | <0.001 |
|  | **NR** | 0 | |  | | 2 | |  | |  |
|  | **Strongly disagree** |  | |  | |  | |  | |  |
|  | **Disagree** |  | |  | |  | |  | |  |
|  | **Neither agree nor disagree** | 5 | | 4.3 | |  | |  | |  |
|  | **Agree** | 27 | | 23.5 | | 6 | | 5.3 | |  |
|  | **Strongly agree** | 83 | | 72.2 | | 107 | | 94.7 | |  |
|  |  |  | |  | |  | |  | |  |
|  | **Median (q1-q3)** | 5 | | 4-5 | | 5 | | 5-5 | |  |
| Q12 |  |  | |  | |  | |  | | <0.001 |
|  | **NR** | 2 | |  | | 1 | |  | |  |
|  | **Strongly disagree** |  | |  | |  | |  | |  |
|  | **Disagree** | 1 | | 0.9 | | 1 | | 0.9 | |  |
|  | **Neither agree nor disagree** | 17 | | 15.0 | | 3 | | 2.6 | |  |
|  | **Agree** | 37 | | 32.7 | | 21 | | 18.4 | |  |
|  | **Strongly agree** | 58 | | 51.3 | | 89 | | 78.1 | |  |
|  |  |  | |  | |  | |  | |  |
|  | **Median (q1-q3)** | 5 | | 4-5 | | 5 | | 5-5 | |  |
| Q13 |  |  | |  | |  | |  | | <0.001 |
|  | **NR** |  | |  | |  | |  | |  |
|  | **Strongly disagree** | 0 | | 0.0 | | 1 | | 0.9 | |  |
|  | **Disagree** | 3 | | 2.6 | | 2 | | 1.7 | |  |
|  | **Neither agree nor disagree** | 17 | | 14.8 | | 8 | | 7.0 | |  |
|  | **Agree** | 34 | | 29.6 | | 12 | | 10.4 | |  |
|  | **Strongly agree** | 61 | | 53.0 | | 92 | | 80.0 | |  |
|  |  |  | |  | |  | |  | |  |
|  | **Median (q1-q3)** | 5 | | 4-5 | | 5 | | 5-5 | |  |
| Q14 |  |  | |  | |  | |  | | <0.001 |
|  | **NR** | 0 | |  | | 1 | |  | |  |
|  | **Strongly disagree** | 4 | | 3.5 | |  | |  | |  |
|  | **Disagree** | 5 | | 4.3 | | 1 | | 0.9 | |  |
|  | **Neither agree nor disagree** | 6 | | 5.2 | | 5 | | 4.4 | |  |
|  | **Agree** | 19 | | 16.5 | | 9 | | 7.9 | |  |
|  | **Strongly agree** | 81 | | 70.4 | | 99 | | 86.8 | |  |
|  |  |  | |  | |  | |  | |  |
|  | **Median (q1-q3)** | 5 | | 4-5 | | 5 | | 5-5 | |  |
| Q15 |  |  | |  | |  | |  | | 0.033 |
|  | **NR** | 0 | |  | | 1 | |  | |  |
|  | **Strongly disagree** | 1 | | 0.9 | |  | |  | |  |
|  | **Disagree** | 2 | | 1.7 | | 2 | | 1.8 | |  |
|  | **Neither agree nor disagree** | 7 | | 6.1 | | 4 | | 3.5 | |  |
|  | **Agree** | 15 | | 13.0 | | 6 | | 5.3 | |  |
|  | **Strongly agree** | 90 | | 78.3 | | 102 | | 89.5 | |  |
|  |  |  | |  | |  | |  | |  |
|  | **Median (q1-q3)** | 5 | | 5-5 | | 5 | | 5-5 | |  |
| Q16 |  |  | |  | |  | |  | | <0.001 |
|  | **NR** | 22 | |  | | 14 | |  | |  |
|  | **Strongly disagree** | 19 | | 20.4 | | 6 | | 5.9 | |  |
|  | **Disagree** | 26 | | 28.0 | | 14 | | 13.9 | |  |
|  | **Neither agree nor disagree** | 31 | | 33.3 | | 37 | | 36.6 | |  |
|  | **Agree** | 8 | | 8.6 | | 18 | | 17.8 | |  |
|  | **Strongly agree** | 9 | | 9.7 | | 26 | | 25.7 | |  |
|  |  |  | |  | |  | |  | |  |
|  | **Median (q1-q3)** | 3 | | 2-3 | | 3 | | 3-5 | |  |
| Q17 |  |  | |  | |  | |  | | 0.002 |
|  | **NR** | 0 | |  | | 3 | |  | |  |
|  | **Strongly disagree** |  | |  | |  | |  | |  |
|  | **Disagree** | 3 | | 2.6 | | 3 | | 2.7 | |  |
|  | **Neither agree nor disagree** | 15 | | 13.0 | | 5 | | 4.5 | |  |
|  | **Agree** | 25 | | 21.7 | | 12 | | 10.7 | |  |
|  | **Strongly agree** | 72 | | 62.6 | | 92 | | 82.1 | |  |
|  |  |  | |  | |  | |  | |  |
|  | **Median (q1-q3)** | 5 | | 4-5 | | 5 | | 5-5 | |  |
| Q18 |  |  | |  | |  | |  | | <0.001 |
|  | **NR** |  | |  | |  | |  | |  |
|  | **Strongly disagree** | 1 | | 0.9 | | 1 | | 0.9 | |  |
|  | **Disagree** | 1 | | 0.9 | |  | |  | |  |
|  | **Neither agree nor disagree** | 4 | | 3.5 | | 4 | | 3.5 | |  |
|  | **Agree** | 29 | | 25.2 | | 8 | | 7.0 | |  |
|  | **Strongly agree** | 80 | | 69.6 | | 102 | | 88.7 | |  |
|  |  |  | |  | |  | |  | |  |
|  | **Median (q1-q3)** | 5 | | 4-5 | | 5 | | 5-5 | |  |
| Q19 |  |  | |  | |  | |  | | 0.121 |
|  | **NR** |  | |  | |  | |  | |  |
|  | **Strongly disagree** | 15 | | 13.0 | | 22 | | 19.1 | |  |
|  | **Disagree** | 23 | | 20.0 | | 20 | | 17.4 | |  |
|  | **Neither agree nor disagree** | 59 | | 51.3 | | 62 | | 53.9 | |  |
|  | **Agree** | 12 | | 10.4 | | 6 | | 5.2 | |  |
|  | **Strongly agree** | 6 | | 5.2 | | 5 | | 4.3 | |  |
|  |  |  | |  | |  | |  | |  |
|  | **Median (q1-q3)** | 3 | | 2-3 | | 3 | | 2-3 | |  |
| Q20 |  |  | |  | |  | |  | | 0.003 |
|  | **NR** |  | |  | |  | |  | |  |
|  | **Strongly disagree** | 30 | | 26.1 | | 49 | | 42.6 | |  |
|  | **Disagree** | 27 | | 23.5 | | 17 | | 14.8 | |  |
|  | **Neither agree nor disagree** | 19 | | 16.5 | | 27 | | 23.5 | |  |
|  | **Agree** | 27 | | 23.5 | | 15 | | 13.0 | |  |
|  | **Strongly agree** | 12 | | 10.4 | | 7 | | 6.1 | |  |
|  |  |  | |  | |  | |  | |  |
|  | **Median (q1-q3)** | 3 | | 1-4 | | 2 | | 1-3 | |  |
| Q21 |  |  | |  | |  | |  | | 0.026 |
|  | **NR** |  | |  | |  | |  | |  |
|  | **Strongly disagree** | 73 | | 63.5 | | 91 | | 79.1 | |  |
|  | **Disagree** | 24 | | 20.9 | | 7 | | 6.1 | |  |
|  | **Neither agree nor disagree** | 11 | | 9.6 | | 10 | | 8.7 | |  |
|  | **Agree** | 6 | | 5.2 | | 6 | | 5.2 | |  |
|  | **Strongly agree** | 1 | | 0.9 | | 1 | | 0.9 | |  |
|  |  |  | |  | |  | |  | |  |
|  | **Median (q1-q3)** | 1 | | 1-2 | | 1 | | 1-1 | |  |
| Q22 |  |  | |  | |  | |  | | 0.010 |
|  | **NR** | 5 | |  | |  | |  | |  |
|  | **Strongly disagree** | 7 | | 6.4 | | 4 | | 3.5 | |  |
|  | **Disagree** | 13 | | 11.8 | | 4 | | 3.5 | |  |
|  | **Neither agree nor disagree** | 19 | | 17.3 | | 26 | | 22.6 | |  |
|  | **Agree** | 32 | | 29.1 | | 28 | | 24.3 | |  |
|  | **Strongly agree** | 39 | | 35.5 | | 53 | | 46.1 | |  |
|  |  |  | |  | |  | |  | |  |
|  | **Median (q1-q3)** | 4 | | 3-5 | | 4 | | 3-5 | |  |
