# Supplementary material for: A nurse-led, telephone-based patient support program for improving adherence in patients with relapsing-remitting multiple sclerosis using interferon beta-1a: Lessons from a consumer-based survey on adveva® PSP
Source: Front Psychol. 2022 Aug 24;13:965229. doi: 10.3389/fpsyg.2022.965229 (PMC9454016; doi:10.3389/fpsyg.2022.965229)
Supplement: Supplementary file 2 [file Table_2.docx]

**Supplementary materials**

**Table 2. Differences among male and female responders in the responses to the survey questions at baselines**

|  |  | Female (n=74) | | Male (n=41) | |
| --- | --- | --- | --- | --- | --- |
|  |  |  | |  | |
|  |  | **n** | **%** | **n** | **%** |
| Q1 |  |  |  |  |  |
|  | **NR** |  |  |  |  |
|  | **Strongly disagree** |  | 0,0 |  | 0,0 |
|  | **Disagree** |  | 0,0 |  | 0,0 |
|  | **Neither agree nor disagree** |  | 0,0 | 1 | 2,4 |
|  | **Agree** | 14 | 18,9 | 7 | 17,1 |
|  | **Strongly agree** | 60 | 81,1 | 33 | 80,5 |
| Q2 |  |  |  |  |  |
|  | **NR** |  |  |  |  |
|  | **Strongly disagree** |  | 0,0 |  | 0,0 |
|  | **Disagree** |  | 0,0 |  | 0,0 |
|  | **Neither agree nor disagree** | 1 | 1,4 | 1 | 2,4 |
|  | **Agree** | 14 | 18,9 | 8 | 19,5 |
|  | **Strongly agree** | 59 | 79,7 | 32 | 78,0 |
| Q3 |  |  |  |  |  |
|  | **NR** |  |  |  |  |
|  | **Strongly disagree** |  |  |  |  |
|  | **Disagree** |  | 0,0 |  | 0,0 |
|  | **Neither agree nor disagree** | 7 | 9,5 | 1 | 2,4 |
|  | **Agree** | 13 | 17,6 | 10 | 24,4 |
|  | **Strongly agree** | 54 | 73,0 | 30 | 73,2 |
| Q4 |  |  |  |  |  |
|  | **NR** |  |  |  |  |
|  | **Strongly disagree** | 1 | 1,4 |  | 0,0 |
|  | **Disagree** | 1 | 1,4 | 1 | 2,4 |
|  | **Neither agree nor disagree** | 16 | 21,6 | 2 | 4,9 |
|  | **Agree** | 16 | 21,6 | 13 | 31,7 |
|  | **Strongly agree** | 40 | 54,1 | 25 | 61,0 |
| Q5 |  |  |  |  |  |
|  | **NR** |  |  |  |  |
|  | **Strongly disagree** |  | 0,0 |  | 0,0 |
|  | **Disagree** |  | 0,0 |  | 0,0 |
|  | **Neither agree nor disagree** | 4 | 5,4 |  | 0,0 |
|  | **Agree** | 13 | 17,6 | 6 | 14,6 |
|  | **Strongly agree** | 57 | 77,0 | 35 | 85,4 |
| Q6 |  |  |  |  |  |
|  | **NR** |  |  |  |  |
|  | **Strongly disagree** |  | 0,0 |  | 0,0 |
|  | **Disagree** | 4 | 5,4 | 1 | 2,4 |
|  | **Neither agree nor disagree** | 4 | 5,4 | 4 | 9,8 |
|  | **Agree** | 21 | 28,4 | 10 | 24,4 |
|  | **Strongly agree** | 45 | 60,8 | 26 | 63,4 |
| Q7 |  |  |  |  |  |
|  | **NR** |  |  |  |  |
|  | **Strongly disagree** |  | 0,0 |  | 0,0 |
|  | **Disagree** |  | 0,0 |  | 0,0 |
|  | **Neither agree nor disagree** | 6 | 8,1 | 2 | 4,9 |
|  | **Agree** | 18 | 24,3 | 11 | 26,8 |
|  | **Strongly agree** | 50 | 67,6 | 28 | 68,3 |
| Q8 |  |  |  |  |  |
|  | **NR** |  |  |  |  |
|  | **Strongly disagree** | 2 | 2,7 |  | 0,0 |
|  | **Disagree** | 1 | 1,4 | 1 | 2,4 |
|  | **Neither agree nor disagree** | 13 | 17,6 | 4 | 9,8 |
|  | **Agree** | 13 | 17,6 | 9 | 22,0 |
|  | **Strongly agree** | 45 | 60,8 | 27 | 65,9 |
| Q9 |  |  |  |  |  |
|  | **NR** | 1 |  |  |  |
|  | **Strongly disagree** | 4 | 5,5 |  | 0,0 |
|  | **Disagree** | 5 | 6,8 | 5 | 12,2 |
|  | **Neither agree nor disagree** | 9 | 12,3 | 6 | 14,6 |
|  | **Agree** | 15 | 20,5 | 11 | 26,8 |
|  | **Strongly agree** | 40 | 54,8 | 19 | 46,3 |
| Q10 |  |  |  |  |  |
|  | **NR** |  |  |  |  |
|  | **Strongly disagree** | 1 | 1,4 |  | 0,0 |
|  | **Disagree** | 4 | 5,4 | 1 | 2,4 |
|  | **Neither agree nor disagree** | 18 | 24,3 | 4 | 9,8 |
|  | **Agree** | 27 | 36,5 | 11 | 26,8 |
|  | **Strongly agree** | 24 | 32,4 | 25 | 61,0 |
| Q11 |  |  |  |  |  |
|  | **NR** |  |  |  |  |
|  | **Strongly disagree** |  | 0,0 |  | 0,0 |
|  | **Disagree** |  | 0,0 |  | 0,0 |
|  | **Neither agree nor disagree** | 4 | 5,4 | 1 | 2,4 |
|  | **Agree** | 18 | 24,3 | 9 | 22,0 |
|  | **Strongly agree** | 52 | 70,3 | 31 | 75,6 |
| Q12 |  |  |  |  |  |
|  | **NR** | 1 |  | 1 |  |
|  | **Strongly disagree** |  | 0,0 |  | 0,0 |
|  | **Disagree** | 1 | 1,4 |  | 0,0 |
|  | **Neither agree nor disagree** | 11 | 15,1 | 6 | 15,0 |
|  | **Agree** | 27 | 37,0 | 10 | 25,0 |
|  | **Strongly agree** | 34 | 46,6 | 24 | 60,0 |
| Q13 |  |  |  |  |  |
|  | **NR** |  |  |  |  |
|  | **Strongly disagree** |  | 0,0 |  | 0,0 |
|  | **Disagree** | 3 | 4,1 |  | 0,0 |
|  | **Neither agree nor disagree** | 12 | 16,2 | 5 | 12,2 |
|  | **Agree** | 20 | 27,0 | 14 | 34,1 |
|  | **Strongly agree** | 39 | 52,7 | 22 | 53,7 |
| Q14 |  |  |  |  |  |
|  | **NR** |  |  |  |  |
|  | **Strongly disagree** | 3 | 4,1 | 1 | 2,4 |
|  | **Disagree** | 3 | 4,1 | 2 | 4,9 |
|  | **Neither agree nor disagree** | 4 | 5,4 | 2 | 4,9 |
|  | **Agree** | 14 | 18,9 | 5 | 12,2 |
|  | **Strongly agree** | 50 | 67,6 | 31 | 75,6 |
| Q15 |  |  |  |  |  |
|  | **NR** |  |  |  |  |
|  | **Strongly disagree** | 1 | 1,4 |  | 0,0 |
|  | **Disagree** | 1 | 1,4 | 1 | 2,4 |
|  | **Neither agree nor disagree** | 5 | 6,8 | 2 | 4,9 |
|  | **Agree** | 9 | 12,2 | 6 | 14,6 |
|  | **Strongly agree** | 58 | 78,4 | 32 | 78,0 |
| Q16 |  |  |  |  |  |
|  | **NR** | 12 |  | 10 |  |
|  | **Strongly disagree** | 11 | 17,7 | 8 | 25,8 |
|  | **Disagree** | 19 | 30,6 | 7 | 22,6 |
|  | **Neither agree nor disagree** | 20 | 32,3 | 11 | 35,5 |
|  | **Agree** | 5 | 8,1 | 3 | 9,7 |
|  | **Strongly agree** | 7 | 11,3 | 2 | 6,5 |
| Q17 |  |  |  |  |  |
|  | **NR** |  |  |  |  |
|  | **Strongly disagree** |  | 0,0 |  | 0,0 |
|  | **Disagree** | 2 | 2,7 | 1 | 2,4 |
|  | **Neither agree nor disagree** | 12 | 16,2 | 3 | 7,3 |
|  | **Agree** | 16 | 21,6 | 9 | 22,0 |
|  | **Strongly agree** | 44 | 59,5 | 28 | 68,3 |
| Q18 |  |  |  |  |  |
|  | **NR** |  |  |  |  |
|  | **Strongly disagree** | 1 | 1,4 |  | 0,0 |
|  | **Disagree** | 1 | 1,4 |  | 0,0 |
|  | **Neither agree nor disagree** | 2 | 2,7 | 2 | 4,9 |
|  | **Agree** | 21 | 28,4 | 8 | 19,5 |
|  | **Strongly agree** | 49 | 66,2 | 31 | 75,6 |
| Q19 |  |  |  |  |  |
|  | **NR** |  |  |  |  |
|  | **Strongly disagree** | 9 | 12,2 | 6 | 14,6 |
|  | **Disagree** | 10 | 13,5 | 13 | 31,7 |
|  | **Neither agree nor disagree** | 41 | 55,4 | 18 | 43,9 |
|  | **Agree** | 8 | 10,8 | 4 | 9,8 |
|  | **Strongly agree** | 6 | 8,1 |  | 0,0 |
| Q20 |  |  |  |  |  |
|  | **NR** |  |  |  |  |
|  | **Strongly disagree** | 18 | 24,3 | 12 | 29,3 |
|  | **Disagree** | 16 | 21,6 | 11 | 26,8 |
|  | **Neither agree nor disagree** | 12 | 16,2 | 7 | 17,1 |
|  | **Agree** | 21 | 28,4 | 6 | 14,6 |
|  | **Strongly agree** | 7 | 9,5 | 5 | 12,2 |
| Q21 |  |  |  |  |  |
|  | **NR** |  |  |  |  |
|  | **Strongly disagree** | 43 | 58,1 | 30 | 73,2 |
|  | **Disagree** | 16 | 21,6 | 8 | 19,5 |
|  | **Neither agree nor disagree** | 10 | 13,5 | 1 | 2,4 |
|  | **Agree** | 4 | 5,4 | 2 | 4,9 |
|  | **Strongly agree** | 1 | 1,4 |  | 0,0 |
| Q22 |  |  |  |  |  |
|  | **NR** | 3 |  | 2 |  |
|  | **Strongly disagree** | 5 | 7,0 | 2 | 5,1 |
|  | **Disagree** | 9 | 12,7 | 4 | 10,3 |
|  | **Neither agree nor disagree** | 15 | 21,1 | 4 | 10,3 |
|  | **Agree** | 24 | 33,8 | 8 | 20,5 |
|  | **Strongly agree** | 18 | 25,4 | 21 | 53,8 |
